# Comparison of doses of Nebulized Magnesium sulphate as an adjuvant treatment with salbutamol in children with Status Asthmaticus

**DOI:** 10.12669/pjms.40.5.7682

**Published:** 2024

**Authors:** Rabia Asif, Heena Rais, Prinka Bai, Ramsha Aziz

**Affiliations:** 1 Rabia Asif, MBBS. Department of Pediatric Medicine, Ziauddin University, Karachi, Pakistan; 2 Heena Rais, FCPS (Pediatric Medicine). Department of Pediatric Medicine, Ziauddin University, Karachi, Pakistan; 3 Prinka Bai, MBBS. Department of Pediatric Medicine, Ziauddin University, Karachi, Pakistan; 4 Ramsha Aziz, MBBS. Department of Pediatric Medicine, Ziauddin University, Karachi, Pakistan

**Keywords:** Asthma, salbutamol, magnesium sulphate, nebulized, Pediatric Rapid Assessment Measure

## Abstract

**Objectives:**

To compare the response between different doses of nebulized magnesium sulphate along with Salbutamol in children between two to 12 years of age with status asthmaticus.

**Methods:**

This single blinded, randomized clinical trial was carried out at the Department of Pediatrics, Dr. Ziauddin University Hospital, Karachi, Pakistan during October 2021 to September 2022. A total of 104 children aged between 2-12 years, with the diagnosis of asthma having “Pediatric Rapid Assessment Measure (PRAM)” score>4 and with reactive airways were included. Children either received three back-to-back nebulization with salbutamol solution only (n=50) or salbutamol and MgSO4 with three different doses (250mg, 500mg or 750mg) after every 20 minutes for 60 minutes. The PRAM score was used as an assessment tool to clinically score asthma.

**Results:**

In a total of 104 children, 53 (51.0%) were girls. The mean age was 5.25±2.86 years. No statistically significant difference was found in PRAM scores at baseline (p=0.448) and at 20-minutes (p=0.072) but significant differences were observed at 40-minutes (p=0.009), 60-minutes (p=0.011), 120-minutes (p=0.010), 6-hours (=0.034), 12-hours (p=0.018), 18-hours (p=0.033) and at 24-hours (p=0.029). The reduction in PRAM scores from baseline to 24-hours following treatment among Salbutamol, Salbutamol+ MgSo4 250mg, Salbutamol+ MgSo4 500mg and Salbutamol+ MgSo4 750mg group were 6.53±1.09, 7.22±1.09, 6.85±1.43 and 7.57±1.06 respectively (p=0.007).

**Conclusion:**

While children with status asthmaticus managed using salbutamol, with or without nebulized MgSO4, showed improved clinical outcomes, combining salbutamol with higher dosages of nebulized MgSO4 resulted in even greater clinical improvement.

***Clinical Trial Registry:*** https://clinicaltrials.gov/ct2/show/NCT04929626

## INTRODUCTION

Asthma is considered as one of the most common chronic illnesses in children. Severe asthma poses a significant social and financial burden on the patients, their families as well as society, with leading cause of multiple admissions and hospital expenses, accounting to more than 1.8 million visits in the emergency department (ED) per annum in the United States.^1^ In Pakistan, prevalence of asthma among children is estimated to be around 20% compared to 10% among adults.^2^ Among children, the prevalence of status asthmaticus is estimated to be 4.5% while overall incidence is calculated to be five per 1000 live-births.^3^

The “Global Initiative for Asthma GINA” guidelines define “asthma exacerbations” (acute asthma or asthma attacks) as “acute or subacute episodes of progressive increase in cough, wheezing, shortness of breath or chest tightness, or combination of these, leading to decreases in expiratory airflow (PEF or FEV1) that can be quantified by measuring lung function”.^4^ The mainstay of treatment for acute asthma includes inhaled short-acting β2-agonists (SABA), nebulized anticholinergic agents, which can prompt synergistic impact with the bronchodilator, in moderate to severe attack of asthma. Corticosteroids are recommended in moderate to severe cases.^5^ Intravenous methyl xanthines like aminophylline are not much effective with a narrow therapeutic range and exert many adverse effects so are only used in severe asthma.^4^

Recent local data has shown the response of salbutamol in 67.4% children with acute excerbation of asthma.^6^ Some researchers propose MgSO4 as a last resort for the treatment in patients not responding to standard treatment and can be used as intravenous or aerosolized form.^7^ The nebulized route has the benefit of rapid onset of action with targeted delivery to the lower respiratory tract and is noninvasive reducing the incidence of adverse effects. Its disadvantages comprise of less dose delivered in contrast to intravenous route and increased respiratory exertion to increase the efficacy of drug.^8^ Aerosolized MgSO4 is very safe, simple to use and cost effective.^9^ Magnesium in infusion form has adverse effects of increasing levels of magnesium in blood, which declines the popularity of IV magnesium sulphate.^2^ So, nebulized MgSO4 was developed to minimize the toxicity of Intravenous form.^9^,^10^

Nevertheless, its use in children in acute severe asthma is uncertain due to scarce information on its use.^2^ However, no specified dose of inhaled magnesium sulphate was used and until now, no specific dose is suggested. So, further studies regarding the dose-response relationship and frequency of administration at different ages are required in both children and adults to identify the exact amount of Magnesium sulphate to be used during the acute asthma attack as inappropriate dosage cannot give the desired response as expected. The objective of this study was to compare the response between different doses of nebulized magnesium sulphate along with Salbutamol in children between two to 12 years of age with status asthmaticus.

## METHODS

This single blinded, randomized clinical trial was carried out at the Department of Pediatrics, Dr. Ziauddin University Hospital (Kemari, Clifton, North campus), Karachi, Pakistan during October 2021 September 2022. Sample size was calculated considering percentage of unexposed with outcome (83.3%) and exposed with outcome (100% ≈ 99%) in previous research done by Kew KM et al. in which they compared good response of magnesium sulphate plus salbutamol group in nebulized form.^11^ We consdired confidence level of 95% and power of test as 80%. The sample size was calculated to be 104.

### Inclusion criteria

Children of either gender between two to 12 years of age, with the diagnosis of asthma having Pediatric Respiratory Assessment Measure (PRAM) score>4 and with reactive airways.

### Exclusion criteria

Critically ill children who required intubation or mechanical ventilation or those who had hypersentivity or allergy to MgSO_4_.

Children with history of chronic lung impairment (as per medical record) and whose parents did not give consent for participation in research were also excluded.

### Ethical Approval

It was taken from the “Institutional Ethical Committee” (Reference: 3490321RAPED, dated: June 7, 2021). Eligible children were enrolled after informed and written consents from them or from their parents / legal guardians.

Patients were randomly divided in two main groups. In Group-A, (n=50), children received three back-to-back nebulization with salbutamol solution only using nebulizer machine and face mask after every 20 minutes for 60 minutes. In Group-B (n=54), children were divided into three sub-groups (18 children in each sub-group) and each subgroup received both salbutamol and MgSO4 with three different doses after every 20 minutes for 60 minutes. First sub-group (n=18) received 250mg (0.5ml) of magnesium sulphate plus salbutamol while second sub-group received 500mg (1.0ml) of MgSO4 along with salbutamol and in third sub-group, 750mg (1.5ml) of MgSO4 along with salbutamol were given. In Group-B, children received salbutamol and MgSO4 (250mg or 500mg or 750mg). Salbutamol nebulizer solution, each nebule contain an isotonic solution of Salbutamol (as sulphate) 2.5mg/2.5ml OR salbutamol respirator solution, of which each ml contains salbutamol as salbutamol sulphate, B.P. 5mg (0.5% w/v). Injection MgSO4 50% w/v, each ml contains MgSO4 7H2O USP 500mg.

PRAM score is used as an assessment tool to clinically score asthma.^4^,^8^ Treatment response in terms of clinical improvement was noted as PRAM Score at baseline and then at 20 minutes interval up to one hour and then at 2^nd^ hour and then after every six hours for 24 hours. Those patients whom clinical condition started to get worse were dropped out from the study and their treatment was stepped up accordingly as other alternate treatment methods for step like injectable magnesium sulphate, terbutaline and ultimately, mechanical ventilation. Duration of hospitalization was also noted. Children were discharged based on their clinical improvement in terms of vital signs and PRAM score less than four and were followed up on outpatient basis. Specially designed proforma was used for data collection.

Data was analyzed using “Statistical Package for Social Sciences (SPSS)” version 26.0. Mean and standard deviation were calculated for numeric variables like age, PRAM score and length of hospitalization. Categorical variables like gender, clinical presentation and drug related side effects were summarized using frequency and percentages. Mean difference of quantitative variables in treatment groups were done using “Analysis of Variance (ANOVA)”. Qualitative data was compared using chi-square test. P-value ≤ 0.05 was considered statistically significant.

## RESULTS

In a total of 104 children, 51 (49.0%) were boys and 53 (51.0%) girls. The mean age was 5.25±2.86 years ranging between 2-12 years. Residential status of all children was urban. Place of admission was emergency room in 84 (80.8%) children while 20 (19.2%) children were enrolled from outpatient department. Past history of asthma was noted in 80 (76.9%) children while 24 (23.1%) children were newly diagnosed cases. Family history of asthma was reported in 67 (64.4%) children. There were 47 (45.2%) children who were using some kinds of asthma preventive medicines while 64 (61.5%) children had history of using salbutamol. Completion of standard pentavalent vaccine was noted in 81 (77.9%) children. Baseline characteristics of children among study groups are shown in Table-I.

**Table-I:** Baseline Characteristics of Children among Study Groups (N=104).

Characteristics		Salbutamol (n=50)	Salbutamol+ MgSo_4_ 250mg (n=18)	Salbutamol+ MgSo_4_ 500mg (n=18)	Salbutamol+ MgSo_4_ 750mg (n=18)	P-Value
Gender	Boys	24 (48.0%)	9 (50.0%)	11 (61.0%)	7 (38.9%)	0.611
	Girls	26 (52.0%)	9 (50.0%)	7 (38.9%)	11 (61.1%)	
Age (years)	2-5	29 (58.0%)	12 (66.7%)	14 (77.8%)	9 (50.0%)	0.045
	6-10	17 (34.0%)	2 (11.1%)	3 (16.7%)	9 (50.0%)	
	11-12	4 (8.0%)	4 (22.2%)	1 (5.6%)	-	
Admission Place	Emergency Room	37 (74.0%)	17 (94.4%)	13 (72.2%)	17 (94.4%)	0.084
	OPD	13 (26.0%)	1 (5.6%)	5 (27.8%)	1 (5.6%)
Newly diagnosed asthma		16 (32.0%)	1 (5.6%)	5 (27.8%)	2 (11.1%)	0.071
Family history of asthma		13 (26.0%)	8 (44.4%)	6 (33.3%)	10 (55.6%)	0.122
Using asthma preventive medicines		22 (44.0%)	11 (61.1%)	8 (44.4%)	6 (33.3%)	0.408
History of Salbutamol Use		28 (56.0%0	15 (83.3%)	10 (55.6%0	11 (61.1%)	0.209
Number of night time asthma symptoms in the past 1 months	None	33 (66.0%)	11 (61.1%)	9 (50.0%)	8 (44.4%)	0.305
	1	13 (26.0)	4 (22.2%)	9 (50.0%)	9 (50.0%)	
	2	2 (4.0%)	1 (5.6%)	-	1 (5.6%)	
	3	2 (4.0%)	2 (11.1%)	-	-
Past history of hospitalization		24 (48.0%)	6 (33.3%)	5 (27.8%)	8 (44.4%)	0.430
Pentavalent Vaccine		40 (80.0%)	12 (66.7%)	17 (94.4%)	12 (66.7%)	0.131
Presenting Symptoms	Fever	20 (40.0%)	12 (66.7%)	6 (33.3%)	9 (50.0%)	0.166
	Cough	50 (100%)	18 (100%)	18 (100%)	18 (100%)	-
	Shortness of breath	48 (96.0%)	18 (100%)	18 (100%)	18 (100%)	0.531
	Lethargy	36 (72.0%)	17 (94.4%)	8 (44.4%0	9 (50.0%)	0.004
	Refusal to Feed	36 (72.0%)	10 (55.6%0	14 (77.8%)	12 (66.7%)	0.488
	Tachycardia	42 (84.0%)	18 (100%)	18 (100%)	100 (100%)	0.025
	Tachypnea	50 (100%)	18 (100%)	18 (100%)	18 (100%)	-
	Chest indrawing	48 (96.0%)	18 (100%)	18 (100%)	18 (100%)	0.531
	Use of accessory muscles	44 (88.0%)	18 (100%)	14 (77.8%)	18 (100%)	0.058
	Cyanosis	3 (6.0%)	-	-	-	0.343
Hemoglobin (g/dl)		10.84±1.52	10.53±1.30	11.29±0.74	11.06±1.34	0.365
TLC		16290±6060	14750±5560	13740±4710	13230±2610	0.119

Comparison of PRAM scores between study groups is shown in Table-II. No statistically significant difference was found in PRAM scores at baseline (p=0.448) and at 20-minutes (p=0.072) but significant differences were observed at 40-minutes (p=0.009), 60-minutes (p=0.011), 120-minutes (p=0.010), 6-hours (=0.034), 12-hours (p=0.018), 18-hours (p=0.033) and at 24-hours (p=0.029). From baseline to 24-hours, reduction in PRAM scores among Salbutamol, Salbutamol+ MgSo4 250mg, Salbutamol+ MgSo4 500mg and Salbutamol+ MgSo4 750mg group were noted to be 6.53±1.09, 7.22±1.09, 6.85±1.43 and 7.57±1.06 respectively (p=0.007).

**Table-II:** Comparison of PRAM Scores during the Course of Study (N=104)

PRAM Scores	Total	Salbutamol (n=50)	Salbutamol+ MgSo_4_ 250mg (n=18)	Salbutamol+ MgSo_4_ 500mg (n=18)	Salbutamol+ MgSo_4_ 750mg (n=18)	P-Value
At 0-minutes	7.47±1.84	7.21±2.03	7.72±1.60	7.13±1.93	7.89±1.49	0.448
At 20-minutes	6.92±1.87	6.46±1.92	7.50±1.76	6.50±2.10	7.50±1.50	0.072
At 40-minutes	5.60±1.77	5.77±1.87	5.89±1.45	4.25±1.53	5.72±1.57	0.009
At 60-minutes	4.58±1.98	4.90±2.26	4.67±1.57	3.19±1.42	4.78±1.06	0.011
At 120-minutes	3.63±1.81	3.85±1.92	3.94±1.51	2.31±1.89	3.89±1.28	0.010
At 6-hours	2.73±1.71	2.79±1.89	3.39±1.42	1.75±1.73	2.78±1.11	0.034
At 12-hours	1.87±1.39	1.75±1.45	2.67±1.24	1.25±1.57	1.94±0.80	0.018
At 18-hours	1.23±1.14	0.98±1.16	1.89±0.32	1.13±1.63	1.33±0.84	0.033
At 24-hours	0.44±0.79	0.68±0.65	0.50±0.51	0.28±0.50	0.32±0.43	0.029

Overall duration of hospitalization was 3.38±1.47 days while comparison of duration of hospitalization showed that Salbutamol+ MgSo_4_ 750mg and Salbutamol+ MgSo_4_ 500mg groups had the lowest duration of hospitalization while difference among the study groups was statistically significant (p<0.001). Comparison of duration of hospitalization among study groups is shown in Table-III.

**Table-III:** Duration of Hospitalization in Study Groups (n=104).

Duration of Hospitalization in days	*Salbutamol (n=50)*	*Salbutamol+ MgSo_4_ 250mg (n=18)*	*Salbutamol+ MgSo_4_ 500mg (n=18)*	*Salbutamol+ MgSo_4_ 750mg (n=18)*	*P-Value*

	4.06±1.56	3.56±0.86	2.57±0.65	2.00±0.77	<0.001

The comparison of drug related side-effects among study groups is shown in Table-IV. No drug related side-effects were reported among children using Salbutamol alone while nausea (p=0.049), vomiting (p=0.021) and respiratory depression (p=0.021) were significantly more among Salbutamol + MgSO_4_ groups.

**Table-IV:** Drug Related Side-Effects in Study Groups (N=104).

Side Effects	Salbutamol (n=50)	Salbutamol+ MgSo_4_ 250mg (n=18)	Salbutamol+ MgSo_4_ 500mg (n=18)	Salbutamol+ MgSo_4_ 750mg (n=18)	P-Value
Nausea	-	2 (11.1%)	-	2 (11.1%)	0.049
Vomiting	-	2 (11.1%)	-	-	0.021
Restlessness	-	1 (5.6%)	2 (11.1%)	2 (11.1%)	0.129
Respiratory depression	-	-	2 (11.1%)	-	0.021

## DISCUSSION

Acute or sub-acute episodes of asthma can present with breathlessness, cough, wheezing and/or chest congestion or any combination of these. In this study, cough, shortness of breath, chest indrawing and lethargy were the most common presentations reported in 100%, 98.1%, 98.1%, and 67.3% children respectively which is consistent with what literature has reported as the most common presenting symptoms.^12^,^13^

The major goal of asthma management is to influence relaxation of airway smooth muscles and reduction in vascular permeability, rise in mucociliary clearance, reduction in mucus secretion and down-regulating the airway inflammatory cycles.^14^ Although, multiple options exist managing acute asthma among children, but most commonly adopted drugs include short-acting β2 agonists such as salbutamol/albuterol or terbutaline. The present study was the first of its kind comparing either salbutamol or combination of salbutamol with three different dosage regiments of MgSO_4_. We found that after 24 hours of treatment, all treatment groups resulted in reduction of PRAM scores but the difference among groups was statistically significant (p=0.029). However, during study intervals, it was found that Salbutamol + MgSO_4_ 500mg resulted in better reduction in PRAM scores at most study intervals. Nannini and colleagues analyzing effectiveness of isotonic MgSO_4_ with salbutamol or salbutamol with normal saline concluded that MgSO_4_ along with salbutamol resulted in better efficacy in terms of resolution of acute asthma clinically.^15^

A study done by Sarhan HA et al showed that nebulized MgSO4 either alone or in combination with salbutamol resulted in significantly better results than salbutamol alone in terms of bronchodilator effects.^16^ A recent study from India compared the effectiveness of nebulized salbutamol with or without MgSO_4_ among children aged between six to 14 years with acute asthma.^17^ The authors found that addition of nebulized MgSO_4_ to salbutamol did not show any significant improvement in clinical effectiveness among children with acute asthma.^17^ A recent study from Bangladesh reported nebulized MgSO_4_ alone to be as effective as nebulized salbutamol among children presenting with acute asthma.^18^ Some other researchers have also shown that a synergistic bronchodilator action could be achieved when salbutamol is used with MgSO_4_.^19^,^20^ We also noted that duration of hospitalization were significantly less among children using the combination of salbutamol and MgSO_4_ when compared to salbutamol alone (p<0.001). A Cochrane review analyzing randomized controlled trials involving MgSO_4_ reported that utilization of nebulized MgSO_4_ in emergency rooms among children with acute asthma reduce the incidence and duration of hospitalization.^21^

In the past, it has been shown that there is no observed therapeutic advantage in incorporating magnesium sulfate into salbutamol nebulization for the management of patients experiencing acute severe symptoms.^22^ Some others have revealed that in patients with status asthmaticus, separate administration of nebulized magnesium sulfate alongside salbutamol showcased bronchodilator responses that were either similar or exhibited lower magnitude and duration compared to salbutamol alone.^23^,^24^ This study’s strengths lie in its rigorous randomized clinical trial design, and exploring various doses of nebulized MgSO4 alongside salbutamol offers insights into dosage impact on clinical outcomes.

### Limitations

It includes relatively small sample size and a single center study place. We did not plan double blinding. We could not monitor and analyze lung functions during the study intervals due to human and resource constraints. Multi-centric studies involving larger set of samples with double blind randomization can further add to what was found in this study.

## CONCLUSION

Although, children with acute asthma managed with salbutamol with or without nebulized MgSO_4_ showed improved clinical outcomes but the combination of salbutamol with nebulized MgSO_4_ at higher dosages resulted in relatively better findings.

### Authors Contribution:

**RA:** Data Collection, Drafting and responsible for the integrity and accuracy of the study.

**HR:** Concept, Methodology, Critical Revisions.

**PB and RA:** Data Analysis, Literature Review.
